# Reconstructing Floorplans from Point Clouds Using GAN

**DOI:** 10.3390/jimaging9020039

**Published:** 2023-02-08

**Authors:** Tianxing Jin, Jiayan Zhuang, Jiangjian Xiao, Ningyuan Xu, Shihao Qin

**Affiliations:** 1 Faculty of Electrical Engineering and Computer Science, Ningbo University, Ningbo 315211, China; 2 Ningbo Institute of Industrial Technology, Chinese Academy of Sciences, Ningbo 315201, China; 3 College of Materials Science and Opto-Electronic Technology, University of Chinese Academy of Sciences, Beijing 100049, China

**Keywords:** point clouds, indoor scene, generative adversarial network (GAN), floorplan reconstruction

## Abstract

This paper proposed a method for reconstructing floorplans from indoor point clouds. Unlike existing corner and line primitive detection algorithms, this method uses a generative adversarial network to learn the complex distribution of indoor layout graphics, and repairs incomplete room masks into more regular segmentation areas. Automatic learning of the structure information of layout graphics can reduce the dependence on geometric priors, and replacing complex optimization algorithms with Deep Neural Networks (DNN) can improve the efficiency of data processing. The proposed method can retain more shape information from the original data and improve the accuracy of the overall structure details. On this basis, the method further used an edge optimization algorithm to eliminate pixel-level edge artifacts that neural networks cannot perceive. Finally, combined with the constraint information of the overall layout, the method can generate compact floorplans with rich semantic information. Experimental results indicated that the algorithm has robustness and accuracy in complex 3D indoor datasets; its performance is competitive with those of existing methods.

## 1. Introduction

Indoor scene planning plays a vital role in scene understanding, reconstruction, and design. Most traditional indoor space plans are drawn manually, which is inefficient, time consuming, and laborious. Currently, it is easy to obtain 3D point clouds of indoor scenes by using a range of equipment such as laser scanners, structured lighting, or stereo vision. However, because indoor scenes contain various furniture items, the point clouds include a large amount of noise and missing data due to occlusion. Making a simple and clear floorplan with an accurate layout from 3D point clouds remains an unsolved problem [1,2].

In the recent past, researchers have reported extensive works on automatic floorplan reconstruction [3,4,5,6,7], and many innovative methods have been proposed. Some methods to indoor layout mapping construct the regional subsets for each room by extracting the set elements of points, lines, and faces from the point clouds [8,9], and describe the shapes of each room by the constraints among the geometric subsets. However, the results obtained by these methods may not be stable because of the difficulty in extracting points, lines, and faces in complex scenes. In addition, most of these algorithms are based on the Manhattan hypothesis, which holds that the floorplan images must be drawn by straight lines parallel to the X-Y plane. In some cases, the generated floorplan cannot provide rich geometric details to meet the needs of accurate reconstruction. Other methods assume that a wall is flat and always perpendicular to the floor. These methods project the point clouds into the floor plane and perform geometric information extraction on the two-dimensional plane, which reduces the complexity of the indoor layout and improves the accuracy and efficiency of prediction. However, the main challenge remains the efficiency and precision of the reconstruction; these algorithms leverage extensive geometric priors and optimization processing, which degrade the robustness and efficiency.

To solve these problems, this paper proposes a novel method to automatically generate fine scene plans from indoor 3D point clouds. The method, proposed in this paper, discards the excessive use of geometric priors and complex geometric optimization by using deep learning instead of traditional methods to obtain a regular and accurate interior geometric layout, and uses optimization algorithms to compensate only for those details that cannot be handled by deep learning. Using deep-learning methods instead of traditional optimization algorithms achieves the highest possible operational efficiency while improving accuracy and precision. Firstly, the method segments the density map into several irregular mask instances using the Mask-RCNN (Region based Convolutional Neural Network) method [10], where each mask instance represents a room. Second, a GAN-based neural network was designed to refine each individual irregular room mask instance to obtain a clear and accurate structure. Finally, by optimizing room boundaries with a layout constraint, this paper generates a tight and accurate room floorplan for the density map. The main contributions of this study are as follows:

(1) The segmentation process by Mask-RCNN divides the overall density map into several room areas, and enlarges each room mask to the same size. It cuts each room into an individual area for further independent optimization and retains the semantic information well for each room. This method can amplify the room features, allowing the model to detect more subtle mask defects, especially in small rooms. At the same time, the zoning method can simplify the input features, prompting the generative model to focus its attention on a single room and improve the repair capability of the generative network.
(2) The proposed method repairs the room mask using the generated network. By introducing a U-Net structure model into the generation network used in this paper, the room instances are regenerated piecewise-fully with a more regular geometric structure. The U-Net structure model can retain more details of the original mask and repair mask defects while preserving as much of the original geometric information as possible.
(3) An edge optimization method is designed to remove those edge artifacts that Convolutional Neural Networks (CNN)-based algorithms cannot handle. It not only makes the mask edges as straight as possible, but also combines the mask instances into a compact and non-overlapping floorplan.
(4) Compared with existing methods, the proposed method offers significant improvements in accuracy and efficiency.

The remaining parts of the paper are as follows. Section 2 discusses related work. Section 3 details the methods of this paper. Section 4 describes the experiment. The conclusion is presented in Section 5.

## 2. Related Work

### 2.1. Floorplan Reconstruction

In traditional methods, a line detection algorithm is used to over-segment 2D space into polygonal facets, and a clustering method is employed to classify and merge the polygonal facets forming simple room instances [11,12]. Those methods depend on the robustness of the line detection algorithm and are sensitive to noise. The introduction of DNNs in recent years has led to significant improvements in both robustness and accuracy. Liu et al. proposed FloorNet to detect keypoints directly for indoor scenes by using the DNN framework with integer programming to generate a floorplan. However, in reality, it is not easy to detect keypoints in scenes with numerous furniture items and complex structures. The incorrect detections may lead to the failure of final floor mapping. With regard to the appeal, Chen et al. proposed Floor-SP to obtain the corner–edge likelihood graph for global energy function minimization by using Mask-RCNN and DNN. In this method, a gradient descent algorithm is also used to estimate the keypoints of the room. It avoids the problem of weak robustness caused by detecting the keypoints directly through DNN. Nevertheless, because the result of Floor-SP comes from secondary reasoning, there is no guarantee that the final result is consistent with the real scene. Stekovic et al. proposed MonteFloor [13], which enhances the layout map reconstruction by introducing Monte Carlo Tree Search, but is computationally inefficient.

### 2.2. Image Generation

GAN, proposed by Goodfellow, uses a discriminator to distinguish the images generated by the generator and guides the discriminator to generate images with specific distributions [14]. However, the original GAN is a process for generating noise input, and it is an undirected generation. Therefore, Pix2pix was proposed for training with paired images [15], causing the network to directionally learn the domain knowledge for picture generation. However, Pix2pix adds random noise to the generator, which makes the network more creative but increases some uncertainty. In addition, the loss function of traditional GAN has certain limitations, which may lead to gradient disappearing when the difference between two domains is large. To overcome this problem, Gulrajani et al. proposed a Wasserstein GAN (WGAN) [16] to measure the similarity between them by using Wasserstein distance, and truncated the weights to solve the problems. However, the weight clipping may cause weight polarization, which can lead to gradient disappearance or gradient explosion. Gulrajani et al. improved WGAN by proposing the use of a gradient penalty, thus solving the problem of gradient disappearance or gradient explosion during training. Nauata et al. proposed house GAN and house GAN++ on the basis of WGAN-GP to generate house layouts; the convolutional message passing neural networks (Conv-MPN) method was also introduced [17,18,19], which allows the room generation model to better incorporate contextual information to generate regular, professional design-aware indoor floorplans. However, these methods are not strongly constrained to accomplish accurate reconstruction.

### 2.3. Instance Segmentation

Mask-based instance segmentation methods include one-stage methods and two-stage methods. The two-stage methods, such as Mask-RCNN, HTC, and PANet [20,21], include an object detection stage and a semantic segmentation stage. These methods use proven object detection methods to generate accurate bounding boxes in the image, and classify the pixels in those bounding boxes. The one-stage methods, such as PolarMask, YOLACT, and SOLO [22,23,24], abandon the object detection stage and obtain the instance mask directly from the image, thus improving the operational efficiency of the model. However, compared with two-stage instance segmentation methods, one-stage methods lose some accuracy. These dense pixel-wise classification methods have achieved great success in the task of instance segmentation, but these algorithms do not account for the geometric contour information of the object, and the performance is degraded on regular geometric objects; in particular, the segmentation effect becomes blurred on the edge details of the object. Unlike these methods, contour-based instance segmentation methods transform instance segmentation into a contour-vertex regression problem. Ling et al. proposed Curve GCN [25], which uses a graph convolution network (GCN) to predict the evolution direction of contour vertices. Peng et al. introduced the snake algorithm into the target detection task; they use the bounding box predicted by center-net as the initial value of the contour, and then obtain instance masks by iterative deformation [26]. Both methods rely on manually designed initial contours, and the large difference between these initial contours and the real contours leads to many inappropriate vertex pairs. To solve this problem, E2EC [27] was proposed, using a learnable contour initialization method to replace the manual initialization method, thereby improving the accuracy of contour prediction. However, these methods are still unable to deal with complex geometries, especially in indoor density maps with noise and defects.

## 3. Method

**Figure 1:**
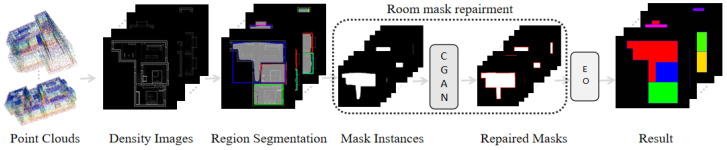
Framework of the proposed method, where CGAN is a GAN-based network and EO is the edge optimization algorithm.

The traditional layout map reconstruction methods based on corner and line segment detection lack robustness in complex indoor spaces. In addition, noise and deficiencies can cause the algorithm to produce incorrect corners or edges, and these incorrect estimates consume a large amount of computation for judgment, which reduces the operational efficiency and reduces the prediction accuracy. To avoid such problems, this paper used generative networks to directly generate masks that can represent the room layout, and obtain accurate and regular vector maps of the indoor layout through simple post-processing. Figure 1 shows the framework of the proposed method, which includes three stages: regional segmentation, room mask repair, and edge optimization.

### 3.1. Region Segmentation

In the first stage, a Mask-RCNN model is used for preliminary regional reconstruction. First, the point clouds are projected to the X-Y plane to generate a density map of 256 × 256 resolution, and then the density map is divided into multiple room instances by using the Mask-RCNN model.

The projection algorithm computes the tight axis-aligned bounding box of the points on the X-Y plane, and the short side extends evenly over both directions to the length of the long side. Then, the projection algorithm extends each of the four directions of the square outwards by 2.5%, and scales the square to a 256 × 256 pixel grid. Finally, pixel values are obtained by counting the number of point clouds corresponding to each pixel grid. The specific calculation formula is as follows:

(1) $$L_{sq}=max*\left(X_{max}-X_{min},Y_{max}-Y_{min}\right),$$

(2) $$P=0.25*L_{sq},$$

(3) $$X_{origin}=X_{min}-P,\;Y_{origin}=Y_{min}+P,\;$$

(4) $$C_{p}=\left(256*\frac{X_{init}-X_{origin}}{L_{sq}+2*P},256*\frac{Y_{origin}-Y_{init}}{L_{sq}+2*P}\right),$$

where $L_{sq}$ is the length of the bounding box. $X_{max}$, $X_{min}$, $Y_{max}$ and $Y_{min}$ are the maximum/minimum values in the point cloud data, and P is the padding width. ($X_{origin}$, $Y_{origin}$) is the coordinate of the origin, and ($X_{init}$, $Y_{init}$) is the original coordinate of the point cloud on the X-Y plane. $C_{p}$ is the pixel coordinate corresponding to the point cloud.

**Figure 2:**
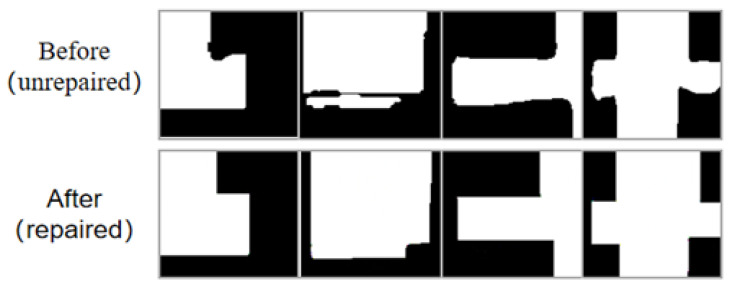
Comparison of unrepaired masks with repaired masks.

Mask-RCNN detects room targets from the image, and then performs semantic segmentation along the room boundary. This paper extracted each room mask from the Mask-RCNN results and scaled them to the same size as the input data for the GAN-based model. In this way, we could obtain the bounding box for each room area and its corresponding contour mask. However, apparent artifacts such as incomplete edges and irregular shapes exist along the room boundary, as shown in the first row in Figure 2. The regional segmentation stage allows the model to focus more on local features, reducing the complexity of the mask geometry and improving the accuracy of the generated masks.

### 3.2. Room Mask Repair Process

Mask-RCNN obtains coarse room masks, which already possess a similar overall structure to the ground truth. However, these masks have many defects that are caused by noise and the algorithm’s own performance. These defects produce incorrect corners and edges that cannot be handled using simple optimization. To improve the mask segments obtained in the previous stage, this stage uses a GAN-based method to repair each incomplete room mask separately. Previous image generation models have more randomness, but the task in this paper needs to preserve the structural information of the original masks, which is more similar to image restoration processing.

**Figure 3:**
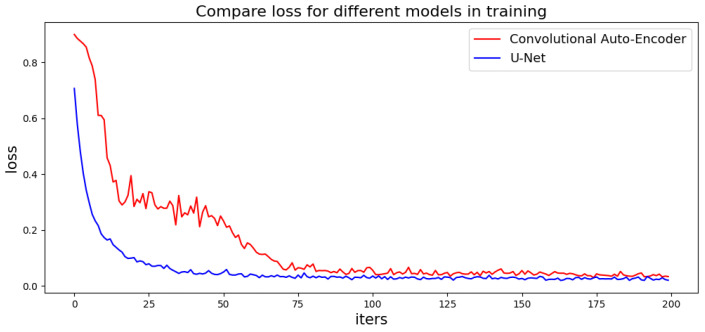
Loss of generators in training.

Traditional GAN-based models use a convolutional auto-encoder as the generator, which has an encoder and a decoder. However, the convolutional auto-encoder loses a lot of information during the encoding and decoding process, making it difficult for training. Here, this paper employed a U-Net [28] structure model with attention mechanisms to build a generation network. U-Net consists of two parts: an encoder and a decoder. The encoder on the left side extracts the features of the image, and the decoder on the right side connects the encoder with the same layer. U-Net up-samples four times, and each time it executes a skip connection, which allows the decoder to retain as much of the original information as possible. Those connections can obtain more accurate multi-scale edge information of the generated images. Figure 3 compares the loss of the U-Net-based generator with the original Convolutional Auto-Encoder in the training stage, where the U-Net-based method exhibits a lower and smoother loss curve. For mask image generation, instead of using original GAN, this paper employed WGAN with Wasserstein distance to measure the difference between the artificial data distribution and real data distribution. The adversarial loss function and the discriminator loss function are defined as

(5) $$L_{wg}=-E_{x\sim P_{f}}\left[D_{w}\left(x\right)\right],$$

(6) $$L_{wd}=E_{x\sim P_{f}}\left[D_{w}\left(x\right)\right]-E_{x\sim P_{r}}\left[D_{w}\left(x\right)\right],$$

where $L_{wg}$ forces the generator to synthesize images close to the real distribution, and $L_{wd}$ is used to enhance the ability of the discriminator to distinguish between true and artificial. Furthermore, L1 loss is added to enhance the learning ability of the generator to create high-quality images at the pixel level. The L1 loss function is defined as

(7) $$L_{l1}\left(G\right)=Mean\left(∥x_{l}-x_{f}{∥}_{1}\right),$$

where $x_{f}$ is the artificial images and $x_{l}$ corresponds to the target images of $x_{f}$. The final generator loss function is formulated as

(8) $$L_{G}=\delta L_{wg}+\partial L_{l1},$$

where δ and ∂ are the weights of the $L_{wg}$ loss function and $L_{l1}$ loss function.

Figure 3 shows the results before and after the GAN repair process, where the repaired mask has a much smoother boundary and the room structure is kept similar to the ground truth. The proposed method can repair various mask defects without complex geometric priors, which improves the efficiency of the algorithm and reduces optimization processes.

### 3.3. Edge Optimization

Even though the structure and boundary of each room are improved significantly after mask repair, some artifacts remain along the room boundaries. In addition, the generated geometric plan is in a non-vector format and lacks semantic information such as wall lines and corners.

**Figure 4:**
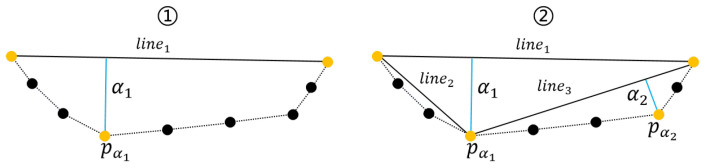
Illustration of the keypoint detection algorithm. Black dots are edge pixel points, yellow dots are keypoints.

Therefore, this paper used a keypoint detection algorithm to obtain keypoints from the repaired masks for room edge extraction. As shown in Figure 4, the algorithm takes two points to obtain a line, and calculate the farthest point to the line between the two points. If the farthest point is less than the threshold farthest point (the keypoint), the algorithm take the keypoint as the midpoint to obtain two lines, then repeat the previous steps until no keypoints are generated. Finally, the algorithm connects the keypoints to obtain edges.

(9) $$\{\begin{array}{c} P_{\alpha \;}\in \;P_{key}\;\;\;\;\;\;\alpha \geq T_{d}\\ None\;\;\;\;\;\;\;\;\;\;\;\;\;\;\;\alpha <T_{d} \end{array},$$

where $p_{\alpha }$ is the farthest point and α is the distance to the line.

**Figure 5:**
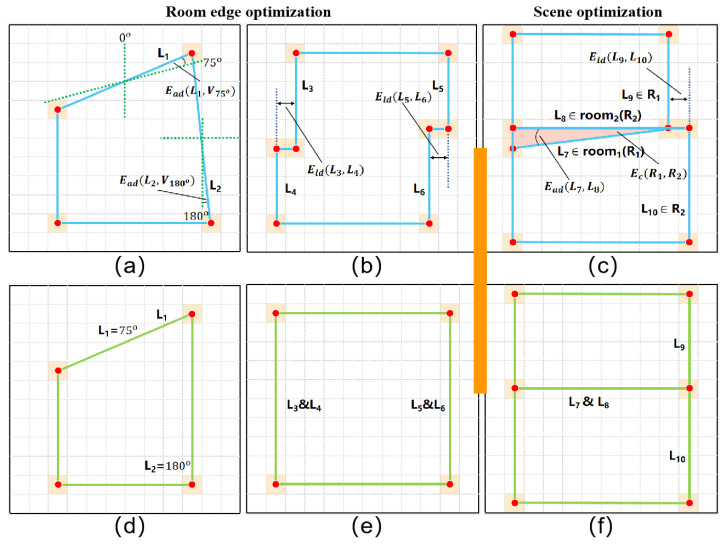
Illustration of the $E_{ad}$, $E_{ld}$, and $E_{c}$ terms. $E_{ad}\;$ is the angle deviation formula, $E_{ld}\;$ is the translation deviation formula, and $E_{c}$ is the coincidence formula. (**a**) and (**b**) indicate defects in individual rooms, (**d**) and (**e**) correspond to the repaired situation. (**c**) and (**f**) indicate before and after overall optimization respectively.

This paper then divide that two-dimensional coordinate system evenly with a rotation scale of 15° (i.e., 0°, 15°, ..., 180°), with left and right symmetry. As shown in Figure 5a,d, when an extracted edge is close to a certain rotation scale, the edge undergoes another small rotation to method this scale. In addition, the parallel edges with close distances are also merged in the same room instance, as illustrated in Figure 5b,e. To obtain the best solution for each room, this paper constructs an energy function for minimization as follows:

(10) $$\begin{array}{c} E_{r}=\sum_{L_{i}\in room}\sum_{V_{t}\in scale}\lambda_{ad}E_{ad}\left(L_{i},V_{t}\right)\\ +\sum_{L_{i}\in room}\sum_{L_{j}\in room}\beta_{ld}E_{ld}\left(L_{i},L_{j}\right) \end{array},$$

where $L_{i}$ and $L_{j}$ are edges of a single room, $V_{t}$ is a scale vector, and $\lambda_{ad}$ is a binary variable. When the angle between the edge and scale is less than the threshold $T_{s\;}$(i.e., 5), $\lambda_{ad}$ is set to 1, otherwise 0. $E_{ad}$ is the angle deflection between the room edge and each rotation scale. By the same token, $\beta_{ld}$ is also a binary variable; when two lines are parallel and close, it is set to 1, otherwise 0.$\;E_{ld}$ is the distance between two edges from a single room.

After optimizing each single room, this paper used the location information of bounding boxes to place the reconstructed room back to the corresponding positions in the floor map for an overall optimization. As shown in Figure 5c, a slight overlap and dislocation exists between neighboring room instances. Therefore, this paper added another energy term $E_{c}$ to penalize the coincidence between each pair of neighboring rooms. Energy item $E_{r}$ was also added to ensure room integrity. The final energy equation is obtained by

(11) $$\begin{array}{c} E_{scene}=\sum_{L_{p}\in scene}\sum_{L_{q}\in scene}(\lambda_{ad}E_{ad}\left(L_{p},L_{q}\right)\\ +\beta_{ld}E_{ld}\left(L_{p},L_{q}\right))+\sum_{R_{i}}\sum_{R_{t}}E_{c}\left(R_{i},R_{t}\right)+E_{r} \end{array},$$

where $L_{p}$ and $L_{q}$ are edges that belong to the whole scene. The item $E_{ad}$ is the angle between edges, and the item $E_{ld}$ is the distance between edges. $\lambda_{ad}$ and $\beta_{ld}$ are binary variables that are activated when the angle or distance between edges is close. The item $E_{c}$ calculates the number of pixel overlaps between rooms.

**Figure 6:**
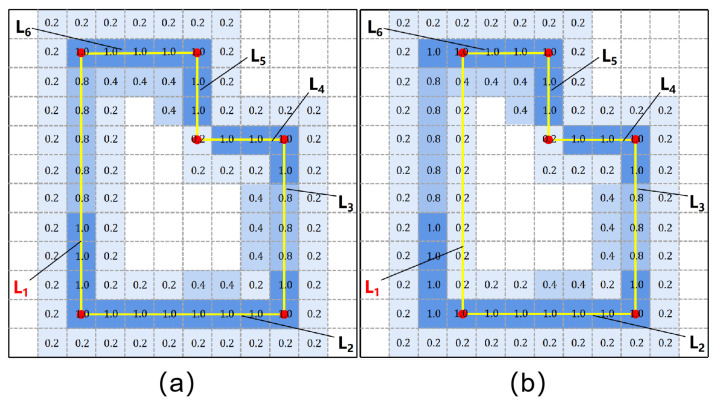
Illustration of the $E_{pix}$ term, which is defined by edge likelihood maps. The energy value is calculated from the pixels that the edge passes through. The energy value in (**a**) is less than the energy value in (**b**).

**Figure 7:**
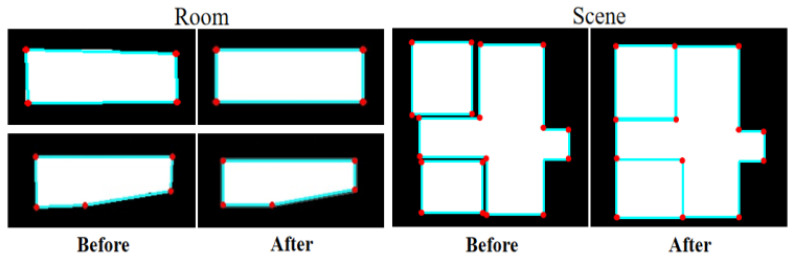
Result of edge optimization.

In addition, a real wall has a certain thickness, and the proposed method assumes that the edge of the floorplan is located in the middle of the wall. Therefore, additional information is needed to constrain the adjustment direction of the edges during the optimization process. The proposed method follows Floor-SP’s edge likelihood map prediction method, which uses the official implementation of Divided Residual Networks to obtain a 256 × 256 edge likelihood map. This paper used the edge likelihood map for geometric constraints. This paper labels the wall edge line as a straight line located in the middle of the wall for training, which ensures that the predicted edge likelihood map has a correct guiding role. As shown in Figure 6, the edge likelihood map describes the likelihood of the existence of edges, which are represented by pixel values. A larger pixel value corresponds with a greater possibility of its belonging to the edge. The proposed method uses this information to construct the energy function and uses the Bresenham algorithm to obtain the pixels corresponding to the edge lines:

(12) $$E_{pix}=\sum_{p_{t}\in L_{e}}p_{t},$$

where $p_{t}$ is one minus the value of the pixels in edge lines. The result of edge optimization, including room and scene, is shown in Figure 7.

## 4. Experiment

### 4.1. Dataset and Setup

The dataset used in the experiment in this study was sourced from the public 3D-Front data of Ali TianChi (https://tianchi.aliyun.com, accessed on 15 April 2021). This paper randomly selected data of 493 simulated indoor scenes (406 scenes as the training dataset and 87 scenes for verification). From each scene, 50,000 points were sampled by uniform sampling with Gaussian noise with a variance of 0.01 added. Then, the point cloud data were projected to the X-Y plane to obtain the density map of each scene. Finally, the professional annotation team was invited to annotate the data collected and obtain the ground truth from specialists.

The GAN-based model uses the RMSprop optimizer with a learning rate of 5 × 10^−4^. The epoch was set to 300, and α and β were set to 0.2 and 0.8, respectively. For Mask-RCNN, the learning rate was set to 1 × 10^−5^ with an epoch of 100. Finally, this paper used the greedy algorithm to solve the edge optimization problem and minimize the energy function.

### 4.2. Qualitative Evaluation

The experiment compared the proposed method with five current advanced methods: ASIP [29], that of Zhang et al. [30], MonteFloor and Floor-SP. ASIP is an object vectorization algorithm, which uses a probability map to generate approximate shapes. The ASIP algorithm trades off complexity and fidelity with a tunable parameter, but there is no systematic method for interpreting or determining this coefficient. Zhang et al.’s algorithm is a recent algorithm that explores and classifies to obtain better results. However, this algorithm lacks regularization, which leads to many defects in the results. One of the state-of-the-art (SOTA) methods known as Floor-SP combines corner detection and edge detection for indoor structure prediction; it has better robustness than FloorNet and can handle non-Manhattan structure scenes. However, Floor-SP uses the shortest path algorithm, and may yield incorrect results in complex nonconvex graphics. MonteFloor is a floorplan reconstruction method that includes a Monte Carlo tree search algorithm, but the search tree operation reduces computational efficiency.

Furthermore, in terms of mask generation, the experiment compared the proposed method (Mask-RCNN+GAN) with the three most advanced methods in the field of instance segmentation, including Mask-RCNN, E2EC, and RefineMask [31]. This paper used vectorization operations on the masks generated by different methods to obtain room plans, and evaluated the precision and recall of the generated corners.

### 4.3. Quantitative Evaluations

This paper defined four metrics for the quantitative evaluations, including the precision, recall and F1-score of corners, edges, and rooms, and overall efficiency.

Corner precision/recall/F1-score: this paper judged the corner to be successfully reconstructed if the ground truth existed within a radius of ten pixels.

Edge precision/recall/F1-score: this paper judged the edge to be successfully reconstructed if both corners of an edge were successfully reconstructed and the edge was real in the ground truth.

Room precision/recall/F1-score: this paper considered a room successfully reconstructed if (1) the room did not overlap with other rooms, and (2) a room whose Intersection over Union (IOU) value of its corresponding ground truth was greater than 0.7.

Efficiency: this paper calculated the total time taken by each method to verify 87 scenes, and compared the efficiency between methods on time taken.

Corner/Edge/Room precision: the precision value is defined as the number of successfully reconstructed corners/edges/rooms divided by the number of all reconstructed corners/edges/rooms.

(13) $$Precision=\frac{Correct\;Reconstruction}{All\;Reconstruction}$$

Corner/Edge/Room recall: the recall value is defined as the number of successfully reconstructed corners/edges/rooms divided by the number of corners/edges/rooms in the ground truth.

(14) $$Recall=\frac{Correct\;Reconstruction}{Ground\;Truth}$$

Corner/Edge/Room F1-score: the F1-score combines precision and recall measurements, as follows:

(15) $$F1-score=2*\frac{Precision*Recall}{Precision+Recall}$$

### 4.4. Experimental Results and Analysis

**Figure 8:**
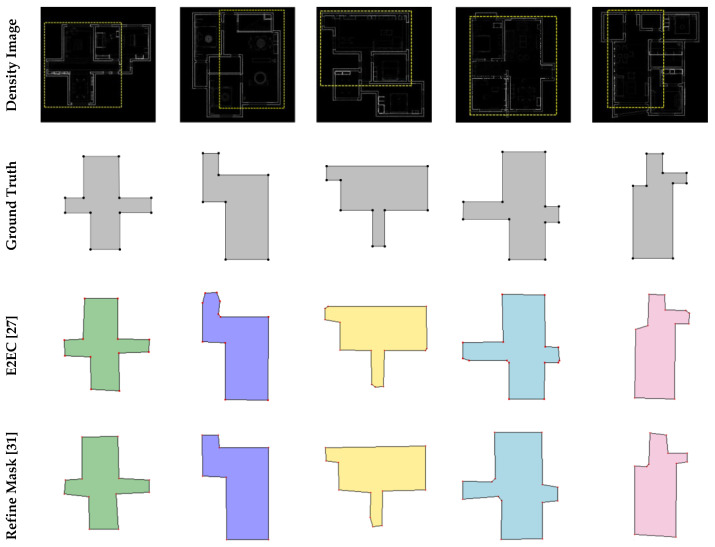

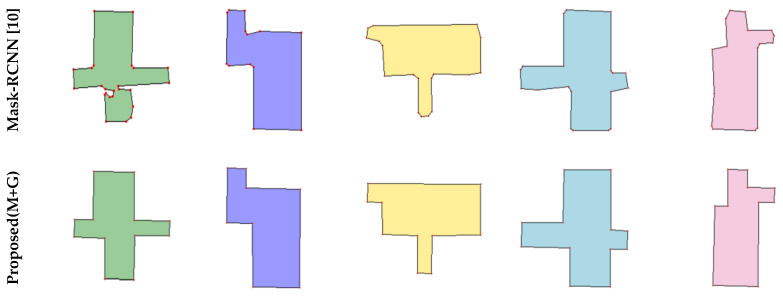
Qualitative comparisons against E2EC, RefineMask, and Mask-RCNN.

**Figure 9:**
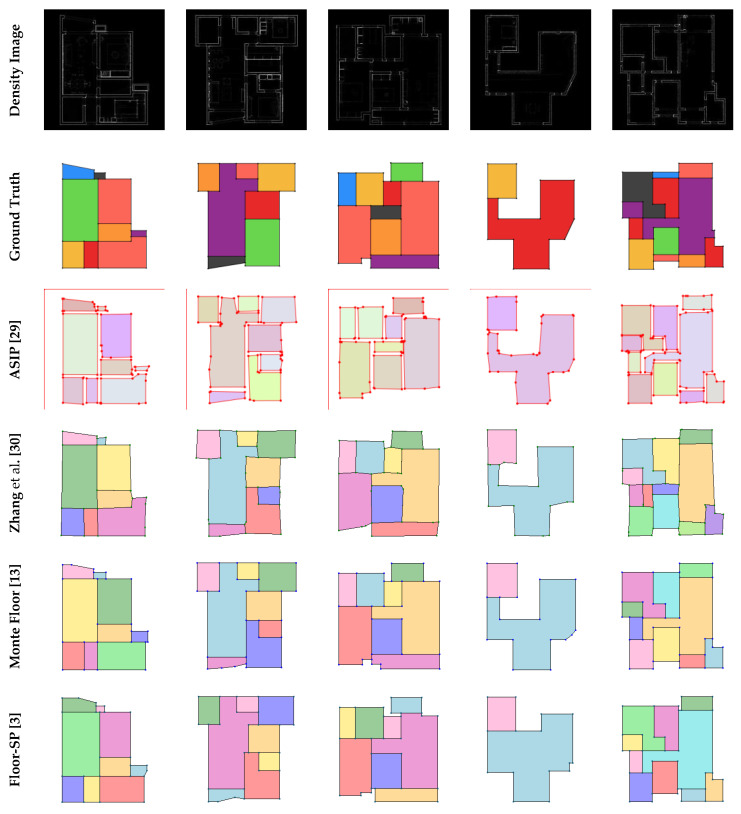

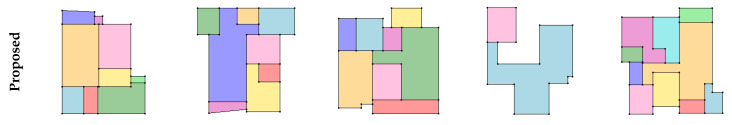
Qualitative comparisons against ASIP, Zhang et al., MonteFloor, and Floor-SP.

There were two main experiments in this paper. The first experiment compared the proposed mask generation method (Mask-RCNN + GAN) with other SOTA instance segmentation methods, thus demonstrating that existing instance segmentation methods cannot meet the requirements of the task. Figure 8 and Table 1 show the results of the first experiment. In order to show more details on masks, the mask of a single room is magnified for comparison in Figure 8. The second experiment was a comparison of the core task in this paper, comparing the proposed method (complete) with advanced methods for reconstructing floorplan. Test results of the second experiment show in Figure 9 and Table 2.

Table 1 shows the results of the comparison between the proposed mask generation method and the other advanced methods. The proposed method achieved the best evaluation result in corner metrics; the precision and recall of corner detection were 0.938 and 0.975, respectively. The precision score of E2EC was high, but the recall score was low, which indicates that E2EC misses many corners in the ground truth. The precision and recall metrics of the other two methods were lower than those of the proposed method, which shows the advantage of the proposed method.

Figure 8 shows that our algorithm outperformed the other methods, because the proposed method uses GAN to repair masks, which eliminates massive incorrect corners and edges. To better show the edge detail of the generated masks, we magnified the single room mask for better comparison. Mask-RCNN obtains masks by classifying pixels, but Mask-RCNN operates without geometric constraints, resulting in incorrect pixel classification. E2EC uses geometric information for mask prediction, but these methods do not place constraints on the shape, resulting in unreasonable edges. RefineMask improves on the traditional instance segmentation algorithm, but is sensitive to noise.

Table 2 shows the results of different methods for four metrics, and indicates the proposed method is superior to the other algorithms in the accuracy of detail reconstruction, including corners and edges. Despite the high computational efficiency of ASIP, the prediction results are poor. In addition, because the proposed method does not perform complex secondary optimization at the pixel level or search tree operation, unlike Floor-SP or MonteFloor, the computational efficiency of the proposed method is significantly improved.

Figure 9 compares the final results obtained by different methods. ASIP can generate rough geometries, but with messy points and edges. Zhang et al.’s algorithm is an improvement over ASIP, but there are still incorrect corner points and edges. As shown in the sixth row of Figure 9, because Floor-SP uses the shortest-path algorithm, it may yield incorrect results in complex non-convex graphics, whereas the proposed method provides a correct result close to the ground truth. MonteFloor gives good results, but there were still some errors in structurally complex regions.

## 5. Conclusions

This paper proposed a novel method to the task of interior reconstruction, introducing a GAN-based method for the task of floorplan reconstruction without using any strong geometric prior constraints. This proposed method can significantly improve the room instances segmented by the traditional Mask-RCNN method. An edge optimization method is further designed to remove edge artifacts along the room boundary, and merge the mask instances into a compact and non-overlapping floorplan. The experimental results demonstrated that the proposed method is competitive in both accuracy and efficiency. In the future, we aim to add automated reconstruction of windows, doors, and furniture to achieve a unified framework for efficient and highly accurate structural reconstruction of indoor scenes using point clouds and panoramic images.

## Figures and Tables

**Table 1:** Test results for different mask generation methods.

		E2EC [27]	Refine Mask [31]	Mask-RCNN [10]	Proposed (M + G)
Corner	Precision	0.959	0.728	0.725	0.938
	Recall	0.757	0.935	0.968	0.975
	F1-score	0.846	0.819	0.829	0.956

**Table 2:** Test results for different floorplan reconstruction methods, and the bold values are the best.

Method	Corner			Edge			Room			Efficiency
	Pre.	Recall	F1-Score	Pre.	Recall	F1-Score	Pre.	Recall	F1-Score	Time (s)
Floor-SP [3]	0.93	**0.97**	0.95	0.93	0.95	0.94	0.93	0.94	0.93	32,667
Zhang et al. [30]	0.90	0.95	0.92	0.85	0.89	0.87	0.92	0.93	0.92	8452
MonteFloor [13]	0.94	0.96	0.95	0.93	0.95	0.94	0.94	0.95	0.94	6237
ASIP [29]	0.83	0.93	0.88	0.75	0.86	0.80	0.91	0.92	0.91	**53**
Proposed	**0.96**	0.97	**0.96**	**0.94**	**0.96**	**0.95**	**0.94**	**0.96**	**0.95**	222

## Data Availability

The data that support the findings of this study are available from the corresponding author, T.J., upon reasonable request.
